# UVB-exposed wheat germ oil increases serum 25-hydroxyvitamin D_2_ without improving overall vitamin D status: a randomized controlled trial

**DOI:** 10.1007/s00394-022-02827-w

**Published:** 2022-02-27

**Authors:** Anja C. Bailer, Sophie Philipp, Shabnam Staudt, Thomas Weidauer, Michael Kiehntopf, Stefan Lorkowski, Gabriele I. Stangl, Christine Dawczynski

**Affiliations:** 1 Institute of Agricultural and Nutritional Sciences, Martin Luther University Halle-Wittenberg, Von-Danckelmann-Platz 2, 06120 Halle (Saale), Germany; 2 Competence Cluster for Nutrition and Cardiovascular Health (nutriCARD) Halle-Jena-Leipzig, Jena, Germany; 3 Junior Research Group Nutritional Concepts, Institute of Nutritional Sciences, Friedrich Schiller University Jena, Dornburger Straße 27, 07743 Jena, Germany; 4 Institute of Clinical Chemistry and Laboratory Diagnostics, University Hospital Jena, 07747 Jena, Germany

**Keywords:** Vitamin D_2_, Vitamin D status, Healthy subjects, Wheat germ oil, UVB-exposure, Randomized controlled trial

## Abstract

**Purpose:**

This study investigated whether UVB-exposed wheat germ oil (WGO) is capable to improving the vitamin D status in healthy volunteers.

**Methods:**

A randomized controlled human-intervention trial in parallel design was conducted in Jena (Germany) between February and April. Ultimately, 46 healthy males and females with low mean 25-hydroxyvitamin D (25(OH)D) levels (34.9 ± 10.6 nmol/L) were randomized into three groups receiving either no WGO oil (control, *n* = 14), 10 g non-exposed WGO per day (– UVB WGO, *n* = 16) or 10 g WGO, which was exposed for 10 min to ultraviolet B-light (UVB, intensity 500–630 µW/cm^2^) and provided 23.7 µg vitamin D (22.9 µg vitamin D_2_ and 0.89 µg vitamin D_3_) (+ UVB WGO, *n* = 16) for 6 weeks. Blood was obtained at baseline, after 3 and 6 weeks and analyzed for serum vitamin D-metabolite concentrations via LC–MS/MS.

**Results:**

Participants who received the UVB-exposed WGO were characterized by an increase of circulating 25(OH)D_2_ after 3 and 6 weeks of intervention. However, the 25(OH)D_3_ concentrations decreased in the + UVB WGO group, while they increased in the control groups. Finally, the total 25(OH)D concentration (25(OH)D_2_ + 25(OH)D_3_) in the + UVB WGO group was lower than that of the non-WGO receiving control group after 6 weeks of treatment. In contrast, circulating vitamin D (vitamin D_2_ + vitamin D_3_) was higher in the + UVB WGO group than in the control group receiving no WGO.

**Conclusion:**

UVB-exposed WGO containing 23.7 µg vitamin D can increase 25(OH)D_2_ levels but do no improve total serum levels of 25(OH)D of vitamin D-insufficient subjects.

**Trial registration:**

ClinicalTrials.gov: NCT03499327 (registered, April 13, 2018).

**Supplementary Information:**

The online version contains supplementary material available at 10.1007/s00394-022-02827-w.

## Introduction

Vitamin D deficiency is widespread among the population worldwide [35, 37]. In times of insufficient endogenous synthesis, e.g. by absent ultraviolet B light (UVB) exposure, recommendations for intake are 15–20 µg vitamin D per day [17, 27]. Vitamin D occurs in two forms in nature, vitamin D_2_ (ergocalciferol) and vitamin D_3_ (cholecalciferol), but food sources of vitamin D are scarce. Relevant amounts of vitamin D are only found in fatty fish [27]. Inefficient endocrine synthesis and inadequate intake of vitamin D result in suboptimal vitamin D status, which is assessed by the analysis of 25-hydroxyvitamin D (25(OH)D), a metabolite of vitamin D which is commonly used as status marker in serum or plasma [40]. Cut-off values indicating insufficient vitamin D status are discussed to be 50 nmol/L [27] or 75 nmol/L [26]. In the U.S. National Health and Nutrition Examination Survey (NHANES) study, the prevalence of individuals who have vitamin D concentrations below 50 nmol/L was 24% [38]. In Europe, the prevalence of insufficient vitamin D concentrations is even higher and is estimated to be 40.4% [9]. The intake of vitamin D supplements is one option to combat vitamin D deficiency [32, 33, 39]. However, vitamin D supplements are not widely used, at least in Germany, and therefore are not suitable to improve vitamin D status in large populations [24]. New food sources of vitamin D could be a more efficient strategy to prevent vitamin D insufficiency. The exposure of foods such as yeast, edible mushrooms or milk to UVB light is a promising approach to increase the vitamin D concentrations in foods and diets [25, 29, 32, 44]. Nowadays, UVB-exposed foods are commercially available and considered to be safe (EFSA Panel on Nutrition, Novel Foods and Food Allergens [14–16].

Plant oils have recently been discovered to be a potential source of vitamin D_2_ and vitamin D_3_ precursors, namely ergosterol and 7-dehydrocholesterol (7-DHC) [4]. In particular, wheat germ oil (WGO) showed relevant concentrations of ergosterol and 7-DHC, ranging from 22.1 to 34.2 µg/g and 0.638 to 0.669 µg/g, respectively. Following a 10 min– UVB-exposure (650 W/cm^2^, in a distance of 15 cm), the vitamin D concentrations increased from non-detectable in the non-treated oil to 1.04 µg/g vitamin D_2_ and 0.037 µg/g vitamin D_3_, respectively. It has been shown that the UVB-exposed WGO was able to significantly raise serum 25(OH)D concentrations in vitamin D-depleted mice [4]. However, data on the bioavailability of vitamin D from UVB-exposed WGO in humans are not yet available. The here presented intervention study aimed to elucidate the potential of UVB-exposed WGO in humans to improve their vitamin D status. The randomized controlled study was conducted in Jena (Germany, 51°N) during February and April in a parallel arm design. Participants who received either no WGO or non-exposed WGO served as control groups. The bioavailability of the vitamin D was assessed by measurements of 25(OH)D serum levels. Oxidation markers in the oils and blood levels of lipids and tocopherols served as safety markers or reference parameters to explain differences in plasma levels of vitamin D metabolites between the groups.

## Materials and methods

### Study design and wheat germ oil

The study protocol has been approved by the Ethics Committee of the Friedrich Schiller University Jena (No. 5417-01/18). The study was registered at clinicaltrials.gov (NCT03499327).

**Fig. 1:**
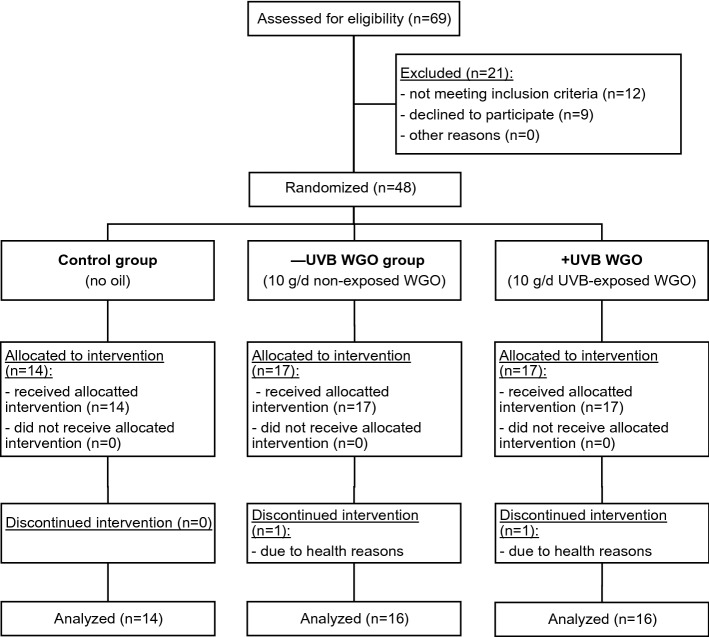
Flow diagram of the participants. Sixty-nine subjects were enrolled in this study. Twenty-one subjects were excluded, since they did not meet the inclusion criteria or declined to participate. Forty-eight subjects were randomized into three groups. As two subjects discontinued the intervention, 46 participants completed the 6 weeks intervention study

The trial was conducted as a randomized controlled study in a three-armed parallel design during February, March and April 2018, when natural sun light intensity in Jena and the surrounding region was low. The intervention period lasted 6 weeks, and the participants were scheduled to visit the study center at baseline and after 3 and 6 weeks. The participants received either no WGO (control, *n* = 14), non UVB-treated wheat germ oil (– UVB WGO, *n* = 17) or UVB-treated WGO (+ UVB WGO, *n* = 17) and were instructed to consume 10 g of the respective oil per day (Fig. 1). The intervention was blinded (except for the control group which received no oil), and participants were not informed about the oil they received. All investigators and physicians were unaware of the group assignment.

The WGO was acquired commercially (vomFass, Waldburg, Germany) and UVB-treated at the Martin Luther University Halle-Wittenberg under food-safe conditions. Therefore, UVB-emitting lamps (UV-15 M, Herolab, Wiesloch, Germany, analyzed intensity 500–630 µW/cm^2^) were placed approximately 19 cm above the oil surface (diameter of the oil surface, 3.5 mm) for 10 min. During UVB-exposure, the oil was constantly stirred by a magnetic stirrer and flushed with nitrogen to avoid oxidation. The oil was UVB-exposed 2 weeks prior to the beginning of the study and had final vitamin D_2_ and vitamin D_3_ concentrations of 2.19 ± 0.36 µg/g and 0.08 ± 0.01 µg/g (*n* = 9), respectively. With a consumption of 10 g oil per day, the participants met their recommended daily intake of vitamin D [27]. The non-exposed WGO was treated, except UVB exposure, in the same way as the UVB-exposed WGO and had vitamin D concentrations below the limit of quantification (LOQ, see chapter 2.4). The participants were instructed to refrigerate the oil during the study period. They were also instructed to consume the not-thermally treated oil pure or e.g., stirred in yogurt.

During each visit, anthropometric data and blood samples were collected for the determination of vitamin D metabolites (vitamin D_2_, vitamin D_3_, 25(OH)D_2_, 25(OH)D_3_), parathyroid hormone (PTH) (primary outcome measures), fatty acid distribution, lipids (triacylglycerols (TAG), total cholesterol (TC), high-density lipoprotein (HDL) cholesterol and low-density lipoprotein (LDL) cholesterol) and tocopherols (secondary outcome measures). The participants documented their normal nutritional habits over 7 days in a food frequency protocol (FFP, originated from PRODI^®^ 5.4 software, Nutri Science, Freiburg, Germany) prior to the baseline and the 6-week visit.

### Subjects

Male and female participants were recruited through newspaper advertisements, information in public institutions and personal contacts in February 2018 in Jena (Germany). Inclusion criteria were: age between 20 and 70 years and serum levels of 25(OH)D < 75 nmol/L. Exclusion criteria were: chronic diseases, medications, consumption of supplements (e.g. vitamin supplements or fish oil capsules), relevant food allergies and visits of sun beds or travel to areas with abundant UVB irradiation during or 3 months prior to the study.

Prior to the study, 69 participants were assessed for eligibility. According to the sample size calculation (see Statistical analysis), a total of 48 participants (age range, 22–66 years) met the inclusion criteria and were randomized in three groups (control, *n* = 14; – UVB WGO, n = 17; + UVB WGO, *n* = 17; Fig. 1). The participants were individually allocated to one of the three study groups, generated by a randomization list with a block size of 8. The allocation ratio of the study oil groups was 1:1.

### Blood collection

After a 12-h fasting period, blood samples were drawn by venipuncture into tubes (Sarstedt, Nümbrecht, Germany). For the analysis of vitamin D metabolites and tocopherols, serum was separated by centrifugation for 10 min at 2000×*g*. For the analysis of PTH, fatty acids and lipids, plasma was separated by centrifugation for 10 min at 1300×*g* and 4 °C and aliquoted. All samples were stored at − 80 °C until further analysis.

### Analytical methods

The concentration of vitamin D_2_ and vitamin D_3_ in WGO was analyzed via liquid chromatography coupled with tandem mass spectrometry (LC–MS/MS). Sample preparation was in accordance with Baur et al. [4]. Analysis was conducted with a QTRAP 5500 MS-system with ESI^+^ ionization (Sciex, Darmstadt, Germany) coupled to a reverse phase HPLC (Agilent 1200, Agilent Technologies, Waldbronn, Germany) equipped with a *Kinetex*^®^* Phenyl-Hexyl* column (100 × 2.1 mm, particle size: 2.6 µm, Phenomenex Incorporation, Torrance, CA, USA). The mobile phase consisted of (A) acetonitrile and (B) aqueous acetonitrile (1/1, v/v) with 5 mmol/L ammonium formate and 0.1% formic acid and a gradient was used for separation (0.0–2.1 min, 85% B; 2.1–7.0 min, 45% B; 18.0 min, 35% B; 22 min, 10% B; 24–26 min, 0% B; 28 min, 100% B; 28.5–30 min, 85%B) with a flow rate of 225 µL/min. MS settings and mass transitions have been reported before [5, 30]. The LOQ was 0.3 µg/g for vitamin D_2_ and 0.03 µg/g for vitamin D_3_. The fatty acid composition of the oils was performed using gas chromatography (GC-17 V3; Shimadzu, Kyoto, Japan) equipped with a flame ionization detector and an autosampler (AOC-5000), as described by Dawczynski et al.[12].

The concentrations of α-, β-, γ- and δ-tocopherol in the WGO were analyzed via LC with fluorescence detector as described before [4]. The acid value was determined according to the German official method [13]. An organoleptic characterization of the oils was conducted by a blinded panel (*n* = 3; ÖHMI Analytik, Magdeburg, Germany). Color, taste, flavor, and viscosity were evaluated [2]. The taste of the oil was ranked by a blinded panel (*n* = 3; ÖHMI Analytik), whereby the best-tasting oil was given the lowest rank (1, 2 or 3) [1]. To rule out the sole influence of the storage period of the WGO, a fresh oil was included as a control.

To analyze the serum concentrations of vitamin D_2_ and vitamin D_3_, the samples were prepared in accordance to Baur et al. [5] and were analyzed by LC–MS/MS as described above for the WGO. The serum concentrations of 25(OH)D_2_ and 25(OH)D_3_ were analyzed via the commercially available *Mass Chrom® 25-OH Vitamin D*_*3*_*/D*_*2*_* reagent kit for LC–MS/MS* (Chromsystems, Gräfelfing, Germany) by use of an Agilent 1200 HPLC system and a QTRAP 5500 MS-System with APCI ionization. The LOQ was 0.25 nmol/L for vitamin D_2_, 1.3 nmol/L for vitamin D_3_, 5.3 nmol/L for 25(OH)D_2_ and 7.5 nmol/L for 25(OH)D_3_.

The concentration of PTH in plasma was analyzed via ELISA according to the manufacturer’s instruction (*Immutopics Human Bioactive PTH 1–84,* TECOmedical, Sissach, Switzerland).

The fatty acid distribution in plasma lipids was analyzed according to Dawczynski et al. [11] by use of gas chromatography with flame ionization detection (GC-17 V3, Shimadzu, Tokyo, Japan), equipped with a fused silica capillary column (DB-225MS, 60 m, inner diameter: 0.25 mm, film thickness: 0.25 µm, Agilent Technologies). Fatty acid concentrations were expressed as percentage of the total area of all fatty acid methyl esters (% of total fatty acid methyl esters, FAME) using GC solution software version 2.3 (Shimadzu).

Plasma lipids (TAG, TC, HDL-cholesterol, LDL-cholesterol) were measured by using an Abbott Architect CI 16,200 analyzer (Abbott, Wiesbaden, Germany) according to the manufacturer’s recommendations.

High performance LC with fluorescent detection was used to analyze the concentration of α-tocopherol in serum as described before [4].

All analyses were run in duplicate.

### Statistical analysis

Sample size calculation was based on previously published data of Seibert et al. [41], who showed an increase in 25(OH)D concentrations from 38 ± 14 to 70 ± 15 nmol/L (Δ25(OH)D, 32 nmol/L; 84% rise) after 8 weeks of supplementation with 20 µg vitamin D_3_ daily during winter time. In contrast, 25(OH)D concentrations in the placebo-treated control group decreased from 38 ± 15 to 32 ± 14 nmol/L. Initially assuming that a daily consumption of 10 g UVB-treated WGO would provide 10 µg vitamin D per day, we hypothesized an increase of 42% to approximately 54 nmol/L in the UVB WGO group. Thus, we calculated a sample size of 14 participants per group with an effect size of 1.47, a 95% power and a significance level of 0.05 (G*Power version 3.1.9.2). To take potential dropouts in the WGO consuming groups into account, 17 participants were included in each WGO group.

Statistical analyses were conducted using SPSS statistics version 24 (IBM, Armonk, NY, USA). For all statistical tests *α* = 0.05 was used to decide if the test result is significant or not. If values were below the LOQ, the appropriate LOQ was used for statistical analysis. To compare the values of the three groups and the absolute changes between baseline and week 6, the data were tested for normal distribution using the Shapiro–Wilk test. Given a normal distribution for all three groups, comparison was done with Welch’s one-way analysis of variance (ANOVA) test. Individual differences were investigated with Games–Howell test. Otherwise, the Kruskal–Wallis test was used and differences between individual groups were investigated using pairwise Mann–Whitney *U* tests with Bonferroni correction.

In case of normal distribution (Shapiro–Wilk test) and equal variances (Mauchly's sphericity test), time-depending differences within a group were compared by repeated measurement ANOVA with post-hoc comparison by Bonferroni. Otherwise, the Friedman test was used and the *P* values were corrected by Bonferroni.

## Results

### Characterization of the wheat germ oils

Since the vitamin D concentrations in UVB-treated WGO are known to increase during storage conditions [4], the concentration of vitamin D in WGO was assessed during the study period. In the non-treated WGO, the concentration of vitamin D (vitamin D_2_ and vitamin D_3_) remained below the LOQ during the whole study period. In the UVB-treated WGO, the vitamin D_2_ concentration ranged from 2.06 to 2.70 µg/g (mean, 2.29 ± 0.18 µg/g, *n* = 9) and the vitamin D_3_ concentration ranged from 0.079 to 0.099 µg/g (mean, 0.089 ± 0.008 µg/g, *n* = 9) (Table 1).

**Table 1:** Characteristics of the wheat germ oils at baseline and at the end of the intervention period

	Control (Fresh WGO)		– UVB WGO		+ UVB WGO	
	Baseline	End	Baseline	End	Baseline	End
Vitamin D, µg/g						
Vitamin D_2_	n.a.		< 0.3	< 0.3	2.34	2.36
Vitamin D_3_	n.a.		< 0.03	< 0.03	0.086	0.099
Total Vitamin D	–		–	–	2.43	2.46
Fatty acids, % FAME						
C16:0 palmitic acid	n.a.		15.2	15.1	15.2	15.1
C18:1 n-9 oleic acid	n.a.		16.5	16.3	16.6	16.2
C18:2 n-6 linoleic acid	n.a.		58.0	58.0	57.8	58.0
C18:3 n-3 α-linolenic acid	n.a.		6.24	6.27	6.26	6.23
Oxidative markers						
Acid value (g KOH/kg)	11.2	11.2	11.2	10.6	11.2	11.0
Tocopherols (mg/100 g)						
α	152	151	151	150	151	149
β	62.2	60.0	61.8	59.3	61.2	58.6
γ	19.8	20.2	20.2	20.6	20.6	20.4
δ	1.47	1.40	1.38	1.37	1.42	1.33
Organoleptic test^a^						
Color	Yellow/Orange, Slightly dull		Yellow/Orange, Slightly dull		Yellow/Orange, Slightly dull	
Aroma	Characteristic		Characteristic		Characteristic	
Flavor	Weak		Moderate		Strong	
Viscosity	Liquid, Slightly viscous		Liquid, Slightly viscous		Liquid, Slightly viscous	
Rank sum^b^	3		6		9	

Data are presented as means of duplicate measures (except for the organoleptic test)

*Fresh WGO* control wheat germ oil, –*UVB WGO* non–UVB exposed wheat germ oil, + *UVB WGO* UVB-exposed wheat germ oil, *n.a.* not analyzed, *FAME* fatty acid methyl esters

^a^The organoleptic test was done five weeks after the UVB-exposure of the oils by a blinded panel (*n* = 3)

^b^Sum of given ranks (1, 2 or 3) by a panel (*n* = 3), with the best-tasting oil receiving the lowest rank

The composition of characteristic fatty acids was similar between the non-treated and UVB-exposed WGOs and did not change during the study period (Table 1). To elucidate, whether the UVB-exposure was accompanied by an increased oxidation of the fatty acids in WGO, the acid value and the concentrations of α-, β-, γ- and δ-tocopherol were analyzed at baseline and at the end of the study period. However, no obvious time- and treatment-dependent differences were observed (Table 1). Organoleptic tests revealed UVB-light induced changes in the flavor of the WGO, because the UVB-exposed WGO achieved the highest number of points, followed by the non-exposed WGO and the fresh WGO (Table 1).

### Baseline characteristics

Forty-eight subjects were enrolled in this study. The baseline characteristics are given in Table 2. Two subjects did not complete the study, due to personal reasons (dropout rate 4.2%). In total, 46 participants (age range, 22–65 years; 19 males/27 females) completed the 6-week-intervention (Fig. 1). The average baseline concentration of 25(OH)D was 35.5 ± 10.4 nmol/L. After the study, the subjects were asked for their compliance in daily oil consumption. From the 32 participants which had to consume the oil daily, eight subjects of the + UVB WGO group (50% of group total) and four subjects of the – UVB WGO group (25% of group total) admitted that they did not consume the oils during 3 or 4 days.

**Table 2:** Baseline characteristics of the study participants

Parameters	Control (no WGO)	– UVB WGO	+ UVB WGO	*P*-value^a^
*n*	14	17	17	-
Age (years)	34 ± 12	34 ± 13	30 ± 5	0.868^a^
Sex (m/f)	3/11	8/9	8/9	-
Weight (kg)	66.9 ± 8.5	70.5 ± 9.0	71.4 ± 12.9	0.417^b^
Body mass index (kg/m^2^)	23.4 ± 2.2	23.4 ± 2.6	22.7 ± 2.5	0.645^b^
Systolic blood pressure (mm Hg)	119 ± 11	124 ± 15	124 ± 16	0.464^b^
Diastolic blood pressure (mm Hg)	79.5 ± 7.0	80.5 ± 8.7	79.1 ± 12.7	0.918^b^
Pulse rate (beats per minute)	72.4 ± 11.8	64.1 ± 8.7	63.9 ± 13.2	0.088^b^

Data are presented as means ± SD

Participants consumed no wheat germ oil (Control), 10 g non–UVB-exposed wheat germ oil (– UVB WGO) or UVB-exposed wheat germ oil (+ UVB WGO) per day

Statistical analysis was conducted by ^a^Kruskal–Wallis or ^b^Welch’s ANOVA

### Nutrient intake assessed by food frequency protocols

The mean daily intake of energy, fat and PUFAs was higher in the two groups which received the WGO (data in Supplementary Table S1). The average daily vitamin D intake was 3.20 ± 2.73 µg vitamin D at baseline and 2.72 ± 2.24 µg at week 6. The vitamin D intake with the background diet (without the WGO) was not different between the groups at any time (Table S1).

### Concentrations of vitamin D status markers

To elucidate the potential of UVB-exposed WGO to improve vitamin D status, the circulating serum concentrations of 25(OH)D were analyzed. At baseline, the 25(OH)D_2_ concentration was below the LOQ in all subjects except one (– UVB WGO group). In subjects treated with the + UVB WGO the concentration of 25(OH)D_2_ increased from baseline to week 3 and 6, respectively (*P* < 0.001 for both time points). The mean level of 25(OH)D_2_ in the control and – UVB WGO groups remained below the LOQ (Table 3). The concentration of 25(OH)D_3_ in the three groups was comparable at baseline and changed during the intervention. The 25(OH)D_3_ levels in the + UVB WGO group decreased steadily during the intervention period, resulting in significant lower levels after 3 and 6 weeks in the + UVB WGO group compared to both other groups (*P* < 0.001 for both time points, Table 3). In contrast, the control and the – UVB WGO group showed a moderate rise in their 25(OH)D_3_ concentrations over the study period (*P* < 0.001 for both groups). The higher concentration of 25(OH)D_3_ in the control and – UVB WGO groups compared to the + UVB WGO group resulted from unusually high 25(OH)D_3_ concentrations analyzed in a few individuals in these groups (three individuals in the control group and three individuals in the – UVB WGO group had 25(OH)D concentrations > 50 nmol/l), whilst other participants had mean values of 29.2 ± 11.0 nmol/L. To elucidate the net effect of the UVB-exposed WGO on vitamin D status, the total 25(OH)D concentrations were calculated by summing up 25(OH)D_2_ and 25(OH)D_3_. Data show that the total 25(OH)D concentrations in the + UVB WGO group remained unchanged, while the concentration of total 25(OH)D moderately increased in the control and in the – UVB WGO groups over the study period (*P* < 0.001 for both groups; Table 3). Finally, the concentration of total 25(OH)D was lower in the + UVB WGO group than in the control group.

**Table 3:** Serum levels of vitamin D status markers at baseline and after 3 and 6 weeks of intervention

	Control (no WGO)	– UVB WGO	+ UVB WGO	*P*-value
*n*	14	16	16	
25(OH)D_2_, nmol/L				
Baseline	< LOQ	< LOQ	< LOQ^z^	n.a
3 weeks	< LOQ^b^	< LOQ^b^	11.6 ± 3.0^a,y^	< 0.001^A^
6 weeks	< LOQ^b^	< LOQ^b^	14.6 ± 3.9^a,x^	< 0.001^B^
Repeated measure analysis	n.a.	n.a.	< 0.001^C^	
25(OH)D_3_, nmol/L				
Baseline	32.7 ± 10.2^y^	28.3 ± 11.5^z,y^	29.9 ± 9.5^x^	0.530^B^
3 weeks	33.2 ± 8.7^a,y^	30.6 ± 13.0^a,y^	21.7 ± 5.6^b,y^	< 0.001^B^
6 weeks	42.3 ± 10.5^a,x^	37.7 ± 15.6^a,x^	20.5 ± 6.1^b,y^	< 0.001^B^
Repeated measure analysis	< 0.001^D^	< 0.001^C^	< 0.001^C^	
Total 25(OH)D, nmol/L				
Baseline	38.0 ± 10.2^y^	33.6 ± 11.6^y^	35.2 ± 9.5	0.535^B^
3 weeks	38.5 ± 8.7^y^	35.9 ± 13.0^y^	33.3 ± 5.7	0.177^B^
6 weeks	47.6 ± 10.5^a,x^	43.0 ± 15.7^ab,x^	35.1 ± 7.2^b^	0.003^B^
Repeated measure analysis	< 0.001^D^	< 0.001^C^	0.829^C^	
Vitamin D_2_, nmol/L				
Baseline	< LOQ	< LOQ	< LOQ^y^	n.a
3 weeks	< LOQ^b^	< LOQ^b^	1.46 ± 0.76^a,x^	< 0.001^A^
6 weeks	< LOQ^b^	< LOQ^b^	1.58 ± 1.05^a,x^	< 0.001^A^
Repeated measure analysis	n.a	n.a	< 0.001^D^	
Vitamin D_3_, nmol/L				
Baseline	1.75 ± 1.22	1.35 ± 0.19^y^	1.85 ± 1.36^y^	0.235^A^
3 weeks	3.05 ± 2.60	2.29 ± 2.69^y^	2.12 ± 0.87^y^	0.220^A^
6 weeks	2.24 ± 1.05^b^	5.29 ± 5.51^ab,x^	5.00 ± 2.44^a,x^	0.002^A^
Repeated measure analysis	0.164^C^	< 0.001^C^	< 0.001^D^	
Total vitamin D, nmol/L				
Baseline	2.00 ± 1.22	1.60 ± 0.19^y^	2.10 ± 1.36^z^	0.235^A^
3 weeks	3.30 ± 2.60^ab^	2.54 ± 2.69^a,y^	3.57 ± 1.23^b,y^	< 0.001^A^
6 weeks	2.49 ± 1.05^b^	5.54 ± 5.51^ab,x^	6.58 ± 2.65^a,x^	< 0.001^A^
Repeated measure analysis	0.164^C^	< 0.001^C^	< 0.001^C^	
Ratio 25(OH)D_2_ to vitamin D_2_				
Baseline	n.a	n.a	n.a.^y^	–
3 weeks	n.a.^b^	n.a.^b^	9.7 ± 4.6^a,x^	< 0.001^A^
6 weeks	n.a.^b^	n.a.^b^	12.5 ± 6.9^a,x^	< 0.001^A^
Repeated measure analysis	–	–	< 0.001^C^	
Ratio 25(OH)D_3_ to vitamin D_3_				
Baseline	22.4 ± 8.9	20.7 ± 7.8^x^	19.6 ± 8.6^x^	0.708^B^
3 weeks	18.1 ± 10.6^ab^	19.0 ± 9.1^a,x^	11.3 ± 3.7^b,x^	0.006^B^
6 weeks	21.0 ± 6.4^a^	12.5 ± 8.2^b,y^	4.6 ± 1.5^c,y^	< 0.001^B^
Repeated measure analysis	0.420^D^	0.005^D^	< 0.001^C^	
Ratio total 25(OH)D to total vitamin D				
Baseline	22.0 ± 7.9	20.8 ± 6.5^x^	19.7 ± 7.6^x^	0.718^B^
3 weeks	17.9 ± 9.7^a^	19.1 ± 8.1^a,xy^	10.2 ± 3.4^b,x^	< 0.001^B^
6 weeks	20.8 ± 5.6^a^	13.0 ± 7.9^b,y^	6.0 ± 2.0^c,y^	< 0.001^B^
Repeated measure analysis	0.374^D^	0.004^D^	< 0.001^C^	
Parathyroid hormone, pmol/L				
Baseline	4.06 ± 4.22^y^	5.87 ± 6.34^y^	4.26 ± 2.71	0.402^A^
3 weeks	5.29 ± 6.72^x^	7.67 ± 10.6^x^	4.33 ± 2.06	0.159^A^
6 weeks	4.71 ± 5.17^xy^	5.64 ± 5.99^y^	5.61 ± 6.88	0.811^A^
Repeated measure analysis	0.046^C^	0.009^C^	0.144^C^	

Data are presented as means ± SD. Participants consumed no wheat germ oil (Control), 10 g non–UVB-exposed wheat germ oil (– UVB WGO) or UVB-exposed wheat germ oil (+ UVB WGO) per day

*LOQ* limit of quantification (25(OH)D_2_, 5.3 nmol/L; vitamin D_2_, 0.25 nmol/L), *25(OH)D* 25-hydroxyvitamin D, *total 25(OH)D* sum of 25(OH)D_2_ and 25(OH)D_3_, *total vitamin D* sum of vitamin D_2_ and vitamin D_3_; *n.a.* not analyzed

^abc^Different superscript letters indicate significant differences between the groups (*P* < 0.05)

^xyz^Different superscript letters indicate significant differences between the appointment times (*P* < 0.05)

Differences between the groups were compared by ^A^Kruskal–Wallis test or ^B^Welch’s ANOVA

Differences between the appointment times were compared by ^C^Friedman test or ^D^ANOVA

In comparison to the 25(OH)D levels, the vitamin D concentrations in serum were noticeably lower. Vitamin D analysis revealed that the circulating concentration of vitamin D_2_ in the + UVB WGO group increased from baseline to week 6 of the study (*P* < 0.001), in contrast to the two other groups. Data on plasma vitamin D_3_ showed a heterogeneous picture. The concentration of vitamin D_3_ rose from baseline to week 6 in the two WGO groups (*P* < 0.001 for both groups), but not in the control group (Table 3). No difference in the final plasma vitamin D_3_ concentration was seen between the + UVB WGO group and the – UVB WGO group. Calculation of the total vitamin D (vitamin D_2_ + vitamin D_3_), revealed a time-dependent rise in in the – UVB WGO and in the + UVB WGO group (*P* < 0.001 for both groups), while the total vitamin D concentration in control group remained unchanged (Table 3). The absolute changes of the vitamin D metabolites after 6 weeks of intervention compared to baseline are given in Table 4.

**Table 4:** Changes in the serum concentrations of hydroxylated and non-hydroxylated vitamin D metabolites after 6 weeks of intervention compared to baseline

	Control	– UVB WGO	+ UVB WGO	*P*-value
*n*	14	16	16	
Δ 25(OH)D_2_ to baseline, nmol/L	n.a	n.a	+ 9.27 ± 3.9	< 0.001
Δ 25(OH)D_3_ to baseline, nmol/L	+ 9.55 ± 6.84^a^	+ 9.43 ± 6.09^a^	− 9.40 ± 8.48^b^	< 0.001
Δ Total 25(OH)D to baseline, nmol/L	+ 9.55 ± 6.84^a^	+ 9.44 ± 6.10^a^	− 0.13 ± 11.2^b^	0.015
Δ Vitamin D_2_ to baseline, nmol/L	n.a.	n.a.	+ 1.33 ± 1.05	< 0.001
Δ Vitamin D_3_ to baseline, nmol/L	+ 0.49 ± 1.15^a^	+ 3.93 ± 5.43^b^	+ 3.15 ± 2.77^b^	0.006
Δ Total vitamin D to baseline, nmol/L	+ 0.49 ± 1.15^a^	+ 3.93 ± 5.43^b^	+ 4.48 ± 3.06^b^	0.001

Participants consumed no wheat germ oil (control), 10 g non-UVB-exposed wheat germ oil (– UVB WGO) or UVB-exposed wheat germ oil (+ UVB WGO) per day

Presented are means ± SD

*25(OH)D* 25-hydroxyvitamin D, *n.a.* not analyzed

Different superscript letters indicate significant differences between the treatment groups (*P* < 0.05).

To elucidate whether the vitamin D source affects the hydroxylation of vitamin D to 25(OH)D, we calculated the ratio of 25(OH)D_2_ to vitamin D_2_, of 25(OH)D_3_ to vitamin D_3_ and of total 25(OH)D to total vitamin D (Table 3). Hydroxylation of vitamin D_3_, and as a result of total vitamin D, was reduced by the daily consumption of both WGOs. However, this effect was much more pronounced in the + UVB WGO than in the – UVB WGO group.

In contrast to vitamin D metabolites, the PTH concentrations did not differ between the groups at any time. After 3 weeks of intervention, the PTH concentrations were higher in the control and in the – UVB WGO groups compared to baseline (*P* < 0.05 and *P* < 0.01, respectively), but not in the + UVB WGO group. However, the PTH concentrations were finally not different after 6 weeks of intervention compared to baseline in all three groups (Table 3).

### Concentrations of plasma lipids

To ensure that changes in serum vitamin D concentrations were not caused by changes in plasma lipids, we analyzed the fatty acid profile, and the plasma concentrations of TAGs and cholesterol. Data show no differences in the profiles of saturated fatty acids (SFAs), monounsaturated fatty acids (MUFAs) and polyunsaturated fatty acids (PUFAs) between the groups after the intervention (Table 5). Since WGO contains high concentrations of linoleic acid (LA), the concentration of LA was analyzed as compliance marker in plasma of the subjects at baseline and after 6 weeks. The plasma LA concentration in the – UVB WGO and the + UVB WGO groups increased from baseline to week 6 of the intervention (Δ baseline vs. week 6: + 1.44 ± 2.61 and + 1.71 ± 2.45% FAME, respectively; *P* < 0.05), while the LA concentration in the control group slightly but not statistically significant declined (Δ: − 0.43 ± 2.22% FAME). Finally, the LA concentration did not differ between the three groups (Table 5). The concentrations of TAGs, total, HDL- and LDL-cholesterol and their ratio were similar between the groups (*P* < 0.1, Table 5).

**Table 5:** Concentrations of fatty acids, lipids, and tocopherol in plasma

	Control (no oil)	– UVB-WGO	+ UVB-WGO	P-value
n	14	16	16	
Σ SFA, % FAME				
Baseline	29.9 ± 2.1^ab^	28.6 ± 2.0^b^	30.7 ± 1.9^a^	0.020^A^
6 weeks	28.4 ± 2.2	28.9 ± 2.3	28.8 ± 3.0	0.675^B^
Σ MUFA, % FAME				
Baseline	25.4 ± 2.9	26.2 ± 2.7	24.9 ± 2.3	0.385^A^
6 weeks	25.6 ± 3.7	25.0 ± 2.2	24.5 ± 2.3	0.630^A^
Σ PUFA, % FAME				
Baseline	41.6 ± 4.2	41.9 ± 3.2	41.1 ± 3.3	0.811^A^
6 weeks	42.8 ± 5.3	43.3 ± 2.6	43.9 ± 3.7	0.801^A^
Linoleic acid, % FAME				
Baseline	29.4 ± 3.7	30.5 ± 3.1	30.2 ± 3.7	0.664^A^
6 weeks	29.0 ± 4.2	32.0 ± 2.7	31.9 ± 3.6	0.076^A^
Triacylglycerols, mmol/L				
Baseline	0.98 ± 0.42	1.02 ± 0.44	0.93 ± 0.30	0.974^B^
6 weeks	1.09 ± 0.71	1.00 ± 0.46	0.95 ± 0.31	0.962^B^
Total cholesterol, mmol/L				
Baseline	4.95 ± 1.08	4.97 ± 1.02	4.59 ± 0.72	0.630^B^
6 weeks	4.99 ± 1.12	4.65 ± 1.10	4.50 ± 0.65	0.732^B^
HDL-cholesterol, mmol/L				
Baseline	1.61 ± 0.38	1.51 ± 0.24	1.62 ± 0.34	0.543^A^
6 weeks	1.72 ± 0.42	1.47 ± 0.27	1.61 ± 0.31	0.134^A^
LDL-cholesterol, mmol/L				
Baseline	2.78 ± 0.82	3.02 ± 0.94	2.61 ± 0.69	0.399^A^
6 weeks	2.74 ± 0.86	2.79 ± 0.94	2.51 ± 0.57	0.531^A^
LDL-cholesterol-/HDL-cholesterol-ratio				
Baseline	1.83 ± 0.69	2.04 ± 0.70	1.71 ± 0.62	0.414^B^
6 weeks	1.69 ± 0.74	1.97 ± 0.83	1.65 ± 0.57	0.388^B^
α-Tocopherol, µg/mL				
Baseline	11.0 ± 3.7	10.7 ± 2.3	9.54 ± 2.1	0.235^A^
6 weeks	10.7 ± 2.5	11.1 ± 3.2	10.3 ± 1.8	0.669^A^

Data are presented as means ± SD. Participants consumed no wheat germ oil (Control), 10 g non–UVB-exposed wheat germ oil (– UVB WGO) or UVB-exposed wheat germ oil (+ UVB WGO) per day

*SFA* saturated fatty acids, *MUFA* monounsaturated fatty acids, *PUFA* polyunsaturated fatty acids, *FAME* fatty acid methyl esters, *HDL* high-density lipoprotein, *LDL* low density lipoprotein

Differences between the groups were compared by ^A^Welch’s ANOVA or ^B^Kruskal–Wallis test

^ab^Different superscript letters indicate significant differences between the groups (*P* < 0.05)

To investigate whether the intake of UVB-treated WGO was accompanied by changes in plasma antioxidants, we analyzed α-tocopherols, but found no time- and group-specific differences (Table 5).

## Discussion

This study investigated the efficacy of UVB-treated WGO to improve the vitamin D status of healthy subjects during the wintertime, when the endogenous vitamin D synthesis is reduced. Firstly, we were able to markedly increase the vitamin D content of WGO by the exposure of this oil to UVB light. The resulting vitamin D content of WGO, which mainly comprised vitamin D_2_, amounted to 2.37 ± 0.16 µg/g (*n* = 9), so that a daily intake of 10 g UVB-treated WGO provided 23.7 µg vitamin D. This intake can be considered as safe, although it is moderately higher (1.6-times) than the recommended daily intake for vitamin D [27]. In addition, the unchanged concentrations of circulating α-tocopherols are not indicative of any oxidative stress associated with the consumption of UVB-treated WGO. However, the UVB-exposure did negatively affect the taste of the oil, as shown by the organoleptic tests.

The major finding of the current study was that UVB-exposed WGO leads to an increase of the serum levels of 25(OH)D_2_, without improving the total 25(OH)D concentrations. The latter resulted from the finding that the treatment with UVB-exposed WGO had lowered 25(OH)D_3_ disproportionately stronger than the treatment with the unexposed WGO. There may be multiple reasons for the strong decline in 25(OH)D_3_ after the consumption of UVB-treated WGO. Firstly, the efficiency of a vitamin D_2_ to increase the serum concentrations of 25(OH)D has been shown to be lower than that of vitamin D_3_ (reviewed in [43]). A few studies which distinguished between the 25(OH)D_2_ and 25(OH)D_3_ concentrations found a marked reduction of 25(OH)D_3_ in vitamin D_2_ treated groups that was stronger than in groups that received no vitamin D [3, 7, 18, 32]. Although both isoforms of vitamin D are considered equally in the treatment of rickets [36], Lehman et al. found substantially lower levels of 25(OH)D in the group supplemented with vitamin D_2_ than in the vitamin D_3_ supplemented group [32]. The lower 25(OH)D levels in the vitamin D_2_ group were caused by a marked decline in 25(OH)D_3_ in comparison to the vitamin D_3_ group. A phenomenon, which has also been demonstrated vice versa [23]. Although the reason for the strong decline of 25(OH)D_3_ concentrations in response to vitamin D_2_ from the UVB-exposed WGO in the present study remains unclear, the data are indicative for a reduced hepatic hydroxylation of vitamin D. This could be due to a competition of vitamin D_2_ and vitamin D_3_ for 25-hydroxylase. Alternatively, the degradation of 25(OH)D_3_ as a result of an upregulated expression of catabolic enzymes by vitamin D_2_ could be enhanced. So far, three enzymes of the cytochrome P450 family (CYP) are known to 25-hydroxylate vitamin D in the liver. While CYP2R1 can hydroxylate both vitamin D isoforms at C-position 25 [46], CYP27A1 is capable of hydroxylating only vitamin D_3_ [21] and CYP3A4 only vitamin D_2_ [22]. The latter is also known to degrade vitamin D_3_, by mono-hydroxylation of 25(OH)D_3_ at several other positions, including C-positions 23, 24, 26 and in particular C4 [45]. Thus, we speculate that the vitamin D_2_ and D_3_ can activate the various hydroxylases in different ways, thereby influencing the 25(OH)D_2_ and 25(OH)D_3_ profile. Secondly, in contrast to semi-synthetic vitamin D_2_ supplements, the UVB treatment of WGO could have resulted in the formation of vitamin D photoisomers such as lumisterol or tachysterol, which in turn may affect the metabolism of the vitamin D_3_ isoforms. In accordance with the current data, UVB-treated mushrooms (providing 17.1 µg vitamin D_2_) were also not able to increase total 25(OH)D levels, since the 25(OH)D_3_ concentrations decreased by 10.3 ± 1.75 nmol/L after 3 weeks and by 20.6 ± 14.6 nmol/L after 6 weeks of intervention [42]. By that, the decrease of 25(OH)D_3_ was higher as the decrease in the current study, which was 8.27 ± 6.19 nmol/L and 9.40 ± 8.48 nmol/L after 3 and 6 weeks, respectively. UVB exposure of food is usually accompanied by the formation of photoisomers. Interestingly, data have shown that vitamin D photoisomers such as lumisterol may lower the circulating 25(OH)D_3_ concentrations in mice [31]. Thus, it is tempting to speculate that availability of vitamin D from UVB exposed food is modified by photoproducts which are synthesized during UVB irradiation. Additionally, the lack of increase in serum 25(OH)D after the consumption of vitamin D_2_ may result from the low dose of administered vitamin D_2_, because higher doses of vitamin D_2_ are capable of increasing total serum concentrations of 25(OH)D_2_. Findings show that high doses of vitamin D_2_ from a UVB-exposed mushrooms soup (700 µg per serving), given as a weekly bolus, can compensate the decrease in 25(OH)D_3_ [10, 44]. It should be pointed out that the 25(OH)D_3_ concentrations increased in the control and – UVB WGO groups, although the participants did not receive any vitamin D, and were encouraged to avoid direct sun exposure and to use sun protection. The observed increase in 25(OH)D_3_ in the control and – UVB WGO groups were indicative of an enhanced endogenous vitamin D synthesis. However, the individual 25(OH)D_3_ levels indicate that the high mean values of 25(OH)D_3_ in the control and – UVB WGO groups were caused by only a few individuals, who do not adhere to the recommendation to avoid sun exposure.

In contrast to the analysis of 25(OH)D, serum levels of vitamin D_2_, did not differ between the – UVB WGO and the + UVB WGO groups, and the – UVB WGO and the control groups, respectively (Table 3). Compared to the control group, the total vitamin D concentrations were higher in the two WGO groups, although the difference was only significant between the + UVB WGO and the control groups, and not between the – UVB WGO and the control groups, due to the high variances. Various compounds are able to modulate the vitamin D metabolism. These are long-chain fatty acids [19] and phytochemicals such as pinoresinol [20] which has been found to lower the intestinal absorption of vitamin D, and ergosterol that appears to increase oral vitamin D_3_ in plasma and liver, of mice [5]. Thus, it is tempting to speculate that the higher vitamin D_3_ concentrations in the – UVB WGO and the + UVB WGO groups observed in the current study are caused by the high amount of ergosterol found in the WGOs. To elucidate, whether the differences in 25(OH)D_3_ were attributed to differences in plasma lipids, we measured plasma fatty acids and other lipids, but found no significant differences between the groups after the intervention. Thus, we suggest that the plasma lipids were not attributable to the observed differences of vitamin D_3_ metabolites. Analysis of plasma PTH revealed no differences between the groups, which indicates the inability of the UVB-treated WGO to improve the vitamin D status.

Our study has several limitations. First, data assume that a few participants had produced vitamin D via the endogenous synthesis although they were asked to avoid any sun light exposure and to use sun protection. Second, the large differences in the response of vitamin D status after vitamin D intake [8, 32], would have required a higher number of study participants to clearly show treatment differences. Third, the male/female ratio was unequally distributed in the groups (control group: males, 3; females, 11; the WGO groups: males, 8; females 8). The higher number of males in the two WGO receiving groups may have resulted in the higher intake of energy, fat and polyunsaturated fatty acids assessed by the FFP [6, 34].

## Conclusion

UVB exposure can significantly increase the vitamin D_2_ concentrations of WGO. The UVB-exposed WGO was able to increase the 25(OH)D_2_ levels in vitamin D insufficient healthy individuals. However, the increase in serum 25(OH)D_2_ was accompanied by a concurrent decrease of 25(OH)D_3_ levels, while the 25(OH)D_3_ levels increased in the control and the – UVB WGO group.

## Supplementary Information

Below is the link to the electronic supplementary material.

Supplementary file1 (DOCX 15 kb)

## Data Availability

Not applicable.

## Abbreviations

25(OH)D: 25-Hydroxyvitamin D
7-DHC: 7-Dehydrocholesterol
ANOVA: Analysis of variance
FAME: Fatty acid methyl esters
FFP: Food frequency protocol
HDL: High-density lipoprotein
LA: Linoleic acid
LDL: Low-density lipoprotein
LOQ: Limit of quantification
MUFA: Monounsaturated fatty acid
PTH: Parathyroid hormone
PUFA: Polyunsaturated fatty acid
SFA: Saturated fatty acid
TAG: Triacylglycerols
TC: Total cholesterol
UVB: Ultraviolet B light
WGO: Wheat germ oil
