# Supplementary material for: UVB-exposed wheat germ oil increases serum 25-hydroxyvitamin D2 without improving overall vitamin D status: a randomized controlled trial
Source: Eur J Nutr. 2022 Feb 27;61(5):2571–83. doi: 10.1007/s00394-022-02827-w (PMC9279215; doi:10.1007/s00394-022-02827-w)
Supplement: Supplementary file 1 — Supplementary file1 (DOCX 15 kb) [file 394_2022_2827_MOESM1_ESM.docx]

**Supplementary Table S1** Daily intake of nutrients in the background diet as evaluated by the food frequency protocols 7-days prior to baseline and week 6

|  | **Control**  **(no oil)** | **-UVB-WGO** | **+UVB-WGO** | **P-value** |
| --- | --- | --- | --- | --- |
| n | 14 | 16 | 16 |  |
| **Energy (kcal)** |  |  |  |  |
| Baseline | 2019 ± 883 | 3045 ± 2844 | 2209 ± 633 | 0.138^1^ |
| 6 weeks | 1850 ± 461^b^ | 2320 ± 714^a^ | 2253 ± 438^a^ | 0.034^1^ |
| **Carbohydrates (g)** |  |  |  |  |
| Baseline | 242 ± 126 | 338 ± 315 | 223 ± 53 | 0.110^1^ |
| 6 weeks | 204 ± 70 | 240 ± 69 | 236 ± 44 | 0.310^2^ |
| **Protein (g)** |  |  |  |  |
| Baseline | 75.6 ± 26.3 | 120 ± 131 | 80.3 ± 27.7 | 0.198^1^ |
| 6 weeks | 70.5 ± 16.1 | 85.4 ± 35.2 | 79.0 ± 16.2 | 0.449^1^ |
| **Fat (g)** |  |  |  |  |
| Baseline | 69.8 ± 31.7^a^ | 114 ± 101^ab^ | 96.9 ± 41.3^b^ | 0.025^1^ |
| 6 weeks | 70.2 ± 23.2^b^ | 100 ± 40^a^ | 94.2 ± 20.0^a^ | 0.013^1^ |
| **SFA (g)** |  |  |  |  |
| Baseline | 27.5 ± 11.6^b^ | 48.7 ± 44.0^ab^ | 42.0 ± 18.1^a^ | 0.016^1^ |
| 6 weeks | 30.2 ± 9.6 | 39.5 ± 14.2 | 37.6 ± 9.0 | 0.115^1^ |
| **MUFA (g)** |  |  |  |  |
| Baseline | 21.7 ± 11.0^b^ | 37.2± 29.0^a^ | 33.5 ± 16.3^a^ | 0.008^1^ |
| 6 weeks | 24.2 ± 9.3 | 32.2 ± 14.9 | 31.2 ± 9.2 | 0.146^1^ |
| **PUFA (g)** |  |  |  |  |
| Baseline | 11.9 ± 8.0 | 15.6 ± 12.4 | 12.6 ± 7.4 | 0.403^1^ |
| 6 weeks | 10.7 ± 5.1^b^ | 21.6 ± 9.9^a^ | 18.7 ± 4.7^a^ | <0.001^1^ |
| **Vitamin D (µg)** |  |  |  |  |
| Baseline | 3.36 ± 2.39 | 3.20 ± 3.16 | 3.07 ± 2.72 | 0.752^1^ |
| 6 weeks | 2.12 ± 1.26 | 3.20 ± 3.03 | 2.77 ± 1.98 | 0.518^1^ |

Data are presented as means ± SD. Participants consumed no wheat germ oil (Control), 10 g non-UVB-exposed wheat germ oil (-UVB WGO) or UVB-exposed wheat germ oil (+UVB WGO) per day. SFA, saturated fatty acids; MUFA, monounsaturated fatty acids; PUFA, polyunsaturated fatty acids. Differences between the groups were compared by ^1^ Kruskal Wallis test or ^2^ Welch’s ANOVA. ^ab^ Different superscript letters indicate significant differences between the groups.
